# Wavelet-based partial volume effect correction for simultaneous MR/PET of the carotid arteries

**DOI:** 10.1186/2197-7364-1-S1-A71

**Published:** 2014-07-29

**Authors:** Jason Bini, Mootaz Eldib, Philip M Robson, Zahi A Fayad

**Affiliations:** Translational and Molecular Imaging Institute, Icahn School of Medicine at Mount Sinai, NY, NY USA; Department of Biomedical Engineering, The City College of New York, NY, NY USA

Simultaneous MR/PET scanners allow for the exploration and development of novel PVE correction techniques without the challenges of coregistration of MR and PET. The development of a wavelet-based PVE correction method, to improve PET quantification, has proven successful in brain PET.^2^ We report here the first attempt to apply these methods to simultaneous MR/PET imaging of the carotid arteries.

The American College of Radiology (ACR) phantom was injected with 18F-FDG for a lesion to background ratio of 2.5. As per ACR protocol, hot cylinders (“lesions”) were injected with 30.71MBq and the phantom background was injected with 12.95MBq. The ACR phantom was then scanned on the Siemens mCT and Siemens Biograph mMR. One patient was injected with 446.6MBq of 18F-FDG and scanned after a circulation time of 90 minutes on the Siemens Biograph mMR. The MR/PET acquisition consisted of the system standard Dixon attenuation correction sequence. Wavelet-based PVE correction was performed to incorporate high frequency wavelet information from MR images into PET images to improve resolution. Qualitative and quantitative uptake parameters where measured to assess the efficacy of the method used.

Qualitative comparisons in the ACR phantom and a patient without and with wavelet-based PVE correction demonstrated slight improvement in image quality. Line profiles drawn through the 8mm cylinder and 25mm cylinder and demonstrated an improvement of quantification in the wavelet-based PVE corrected PET image compared to the non-corrected PET image. Standard deviation of SUV before and after PVE correction in the left and right carotid arteries was decreased demonstrating partial volume effect correction.

The technique applied here demonstrated an improvement in both resolution and quantification in the phantom and the patient. These results demonstrate the feasibility of wavelet-based PVE correction to provide improved quantification for MR/PET in the carotid arteries.

